# Trait impulsivity in Juvenile Myoclonic Epilepsy

**DOI:** 10.1002/acn3.51255

**Published:** 2020-12-02

**Authors:** Amy Shakeshaft, Naim Panjwani, Robert McDowall, Holly Crudgington, Javier Peña Ceballos, Danielle M. Andrade, Christoph P. Beier, Choong Yi Fong, Joanna Gesche, David A. Greenberg, Khalid Hamandi, Jeanette Koht, Kheng Seang Lim, Alessandro Orsini, Mark I. Rees, Guido Rubboli, Kaja K. Selmer, Anna B. Smith, Pasquale Striano, Marte Syvertsen, Inga Talvik, Rhys H. Thomas, Jana Zarubova, Mark P. Richardson, Lisa J. Strug, Deb K. Pal, the BIOJUME Consortium

**Affiliations:** ^1^ Department of Basic & Clinical Neurosciences Institute of Psychiatry, Psychology & Neuroscience King’s College London UK; ^2^ MRC Centre for Neurodevelopmental Disorders King’s College London UK; ^3^ The Hospital for Sick Children Toronto Canada; ^4^ Toronto Western Hospital Canada; ^5^ Odense University Hospital Odense Denmark; ^6^ Division of Paediatric Neurology Department of Paediatrics Faculty of Medicine University of Malaya Kuala Lumpur Malaysia; ^7^ Nationwide Children’s Hospital Ohio; ^8^ Cardiff & Vale University Health Board UK; ^9^ Department of Neurology Drammen Hospital Vestre Viken Health Trust Oslo Norway; ^10^ University of Oslo Oslo Norway; ^11^ Division of Neurology Department of Medicine Faculty of Medicine University of Malaya Kuala Lumpur Malaysia; ^12^ Department of Clinical & Experimental Medicine Pisa University Hospital Italy; ^13^ Neurology Research Group Swansea University Medical School UK; ^14^ Danish Epilepsy Centre Dianalund Denmark; ^15^ University of Copenhagen Denmark; ^16^ Department of Research and Innovation Division of Clinical Neuroscience Oslo University Hospital Norway; ^17^ National Centre for Epilepsy Oslo University Hospital Norway; ^18^ IRCCS Istituto 'G. Gaslini' Genova Italy; ^19^ University of Genova Genova Italy; ^20^ Tallin Children’s Hospital Tallin Estonia; ^21^ Newcastle upon Tyne NHS Foundation Trust Newcastle UK; ^22^ Department of Neurology Motol University Hospital Prague Czech Republic; ^23^ Second Faculty of Medicine Charles University Prague Czech Republic; ^24^ King's College Hospital London UK; ^25^ Evelina London Children's Hospital London UK

## Abstract

**Objective:**

Impulsivity is a multidimensional construct that can predispose to psychopathology. Meta‐analysis demonstrates an association between response impulsivity and Juvenile Myoclonic Epilepsy (JME), a common genetic generalized epilepsy. Here, we test the hypotheses that trait impulsivity is (i) elevated in JME compared to controls; (ii) moderated by specific seizure characteristics; and (iii) associated with psychiatric adverse effects of antiepileptic drugs (AEDs).

**Methods:**

322 participants with JME and 126 age and gender‐matched controls completed the Barratt’s Impulsiveness Scale (BIS‐brief) alongside information on seizure history and AED use. We compared group BIS‐brief scores and assessed associations of JME BIS‐brief scores with seizure characteristics and AED adverse effects.

**Results:**

The mean BIS‐brief score in JME was 18.1 ± 4.4 compared with 16.2 ± 4.1 in controls (*P* = 0.0007). Elevated impulsivity was associated with male gender (*P* = 0.027), frequent absence seizures (*P* = 0.0004) and lack of morning predominance of myoclonus (*P* = 0.008). High impulsivity significantly increased the odds of a psychiatric adverse event on levetiracetam (*P* = 0.036), but not any other psychiatric or somatic adverse effects.

**Interpretation:**

Trait impulsivity is elevated in JME and comparable to scores in personality and neurotic disorders. Increased seizure frequency and absence of circadian seizure pattern moderate BIS score, suggesting disruption of both cortico‐striatal and thalamocortical networks as a shared mechanism between seizures and impulsivity in JME. These findings warrant consideration of impulsivity as a distinct target of intervention, and as a stratifying factor for AED treatment in JME, and perhaps other types of epilepsy. The role of impulsivity in treatment adherence and psychosocial outcome requires further investigation.

## Introduction

Impulsivity is a notable feature of Juvenile Myoclonic Epilepsy (JME), a common genetic epilepsy with complex inheritance.^1^ JME is characterized by adolescent‐onset, generalized seizures, occurring shortly after awakening, and often triggered by sleep fragmentation. Frontal lobe anatomy and function, thalamocortical drive and cortico‐striatal connectivity are altered in JME.^2^, ^3^, ^4^, ^5^, ^6^ Early descriptions emphasized emotional instability and unreliability in patients,^7^ later linked to Cluster B personality disorders.^8^ We have recently shown reduced response inhibition, a dimension of impulsivity, in a meta‐analysis of 1,047 JME patients^9^; however, evidence for impairment in other dimensions of impulsivity, such as choice^5^, ^10^, ^11^, ^12^, ^13^, ^14^ and trait impulsivity,^10^, ^12^, ^13^, ^15^ are scarce or arise from small, uncontrolled studies. The characterization of these discrete dimensions of impulsivity (response, choice and trait) is necessary, as they each contribute to distinct psychiatric phenotypes.^16^, ^17^ Furthermore, the clinical utility of antiepileptic drugs (AEDs), such as levetiracetam, in JME is limited by psychiatric adverse effects, which highly impulsive individuals or those with comorbid psychiatric disorders may be predisposed to ^18^, ^19^, ^20^, ^21^.

Here, we address three issues with major clinical implications in a large, cross‐sectional JME cohort. First, whether trait impulsivity is elevated in JME compared to age and gender‐matched controls. If confirmed, this may justify the assessment and management of impulsivity as a distinct therapeutic target, and motivate the investigation of associations with treatment adherence^22^, ^23^ and long‐term social prognosis.^24^, ^25^ Second, an exploration of moderators of impulsivity, where we test the hypothesis that trait impulsivity is positively associated with seizure characteristics. Moderators may indicate mechanisms and potential interventions for impulsivity in epilepsy. Third, we test the hypothesis that psychiatric, but not somatic, adverse effects of AEDs are associated with elevated trait impulsivity. These findings would assist clinical prediction models of adverse effects and motivate the search for genetic mediators in precision medicine.^26^, ^27^

## Methods

### Participants and data collection

We collected cross‐sectional data as part of the Biology of Juvenile Myoclonic Epilepsy (BIOJUME) genome‐wide association consortium study across 50 sites in 10 countries (appendix). Inclusion criteria were based on Avignon Class II consensus criteria for JME diagnosis^28^:

- Age of myoclonus onset 6‐25 years
- Myoclonic seizures with predominant/exclusive early‐morning pattern involving upper extremities
- Electroencephalogram (EEG) showing interictal generalized spikes and/or polyspike and waves on a normal background
- Current age between 6‐55 years.

Exclusion criteria:

- Myoclonic seizures only associated with carbamazepine or lamotrigine therapy
- EEG showing predominant focal interictal epileptiform discharges or abnormal background
- Evidence of progressive or symptomatic myoclonus epilepsy or focal seizures
- Global learning disability
- Dysmorphic syndrome
- Unable to provide informed consent.

Research staff collected clinical data face‐to‐face in the form of a structured questionnaire, augmented by clinical records, EEG reports, and digital EEGs. The dataset included general demographics and health information, epilepsy history including seizure types, seizure frequency, drug/lifestyle interventions, and BIS‐brief (Table 1).

**Table 1:** Clinical details of participants, including comparison between males and females.

Variable	Total	Female	Male	*P* value	Test type
Total Number	322 (100%)	216 (67.1%)	106 (32.9%)	N/A	N/A
Median age at JME diagnosis (y, range)	16 (6‐40)	15 (6‐37)	16 (9‐40)	0.12	Mann–Whitney
Median JME duration (y, range)	6.2 (0‐39.4)	6.6 (0‐39.4)	4.9 (0‐30.3)	0.24	Mann–Whitney
Median age (y, range)	24 (11‐53)	24 (11‐53)	23 (13‐46)	0.91	Mann–Whitney
Median BMI (kg/m^2^ range)	23.7 (15‐52)	23.5 (15‐52)	25.2 (15‐44)	0.21	Mann–Whitney
BMI category					
Underweight (<18.5)	25 (8.9%)	17 (8.8%)	8 (9.3%)	0.88	Chi squared
Healthy (18.5‐24.9)	134 (47.9%)	100 (51.5%)	34 (39.5%)	0.06	Chi squared
Overweight (25‐29.9)	75 (26.8%)	50 (25.8%)	25 (29.1%)	0.57	Chi squared
Obese (>30)	46 (16.4%)	27 (13.9%)	19 (22.1%)	0.09	Chi squared
Seizure types					
Myoclonus only	17 (5.4%)	11 (5.1%)	6 (5.9%)	0.83	Chi squared
Myoclonus and GTCS	147 (46.5%)	94 (43.7%)	53 (52.5%)	0.27	Chi squared
Myoclonus and absence	20 (6.3%)	17 (7.9%)	3 (3.0%)	0.08	Chi squared
Myoclonus, absence and GTCS	132 (41.8%)	93 (43.3%)	39 (38.6%)	0.28	Chi squared
Mean age of myoclonus onset (y, SD)	14.7 (3.00)	14.5 (2.94)	15.1 (3.05)	0.13	T‐test
Prediagnostic frequency of myoclonus					
Daily	73 (33.3%)	53 (34.4%)	20 (30.8%)	0.60	Chi squared
Weekly	77 (35.2%)	53 (34.4%)	24 (36.9%)	0.72	Chi squared
Less than weekly	69 (31.5%)	48 (31.2%)	21 (32.3%)	0.87	Chi squared
Current frequency of myoclonic seizures					
Daily	39 (12.4%)	26 (12.2%)	13 (12.7%)	0.89	Chi squared
Weekly	51 (16.2%)	29 (13.6%)	22 (21.6%)	0.07	Chi squared
Less than weekly	174 (55.2%)	124 (58.2%)	50 (49.0%)	0.13	Chi squared
None	51 (16.2%)	34 (16.0%)	17 (16.7%)	0.87	Chi squared
Morning predominance of myoclonus	237 (76.9%)	161 (78.2%)	76 (74.5%)	0.48	Chi squared
Absence seizures	153 (48.3%)	111 (51.4%)	42 (41.6%)	0.10	Chi squared
Median absence age of onset (y, range)	13 (3‐37)	12 (3‐37)	14 (8‐30)	**0.03**	Mann–Whitney
Current frequency of absence seizures					
Daily	24 (7.8%)	17 (8.0%)	7 (7.2%)	0.81	Chi squared
Weekly	23 (7.4%)	18 (8.5%)	5 (5.2%)	0.30	Chi squared
Less than weekly	81 (26.2%)	59 (27.8%)	22 (22.7%)	0.34	Chi squared
Currently none	17 (5.5%)	13 (6.1%)	4 (4.1%)	0.47	Chi squared
None ever	164 (53.1%)	105 (49.5%)	59 (60.8%)	0.07	Chi squared
GTCS	282 (88.4%)	187 (87.0%)	95 (91.3%)	0.25	Chi squared
Median age of GTCS onset (y, range)	15 (5‐37)	15 (5‐37)	15 (12‐31)	0.18	Mann–Whitney
Median number of prediagnostic GTCS	2 (0‐200)	2 (0 ‐ 50)	1 (0‐200)	0.17	Mann–Whitney
Lifetime AED therapy					
Valproate	202 (62.7%)	118 (54.6%)	84 (79.2%)	**0.000018**	Chi squared
Lamotrigine	199 (61.8%)	152 (70.4%)	47 (44.3%)	**6.0 x 10** ^‐6^	Chi squared
Levetiracetam	185 (57.5%)	147 (68.1%)	38 (35.8%)	**4.0 x 10** ^‐8^	Chi squared
Topiramate	47 (14.6%)	36 (16.7%)	11 (10.4%)	0.13	Chi squared
None of the above	6 (1.9%)	4 (1.9%)	2 (1.9%)	0.98	Chi squared
Median number of AEDs used (range)	2 (0‐4)	2 (0‐4)	1 (0‐4)	**0.001**	Mann–Whitney
History of AED side effects					
Weight gain on valproate	66 (33.3%)	47 (40.2%)	19 (23.5%)	**0.014**	Chi squared
Psychiatric event to levetiracetam	68 (37.2%)	51(34.9%)	17 (45.9%)	0.22	Chi squared
Skin rash withdrawal on lamotrigine	15 (7.6%)	10 (6.7%)	5 (10.6%)	0.37	Chi squared
Cognitive adverse event to topiramate	6 (13.6%)	4 (11.8%)	2 (20.0%)	0.51	Chi squared
Psychiatric adverse event to topiramate	3 (6.7%)	1 (2.9%)	2 (18.2%)	0.14	Chi squared
Weight loss on topiramate	16 (35.6%)	12 (35.3%)	4 (36.4%)	0.95	Chi squared
Mean BIS‐brief score (SD)	18 .1 (4.4)	17.7 (4.5)	18.8 (4.2)	**0.043**	T‐test

Note

Bold p‐values are significant at *P* < 0.05.

Abbreviations: AED, antiepileptic drug; BMI, Body Mass Index; GTCS, generalized tonic‐clonic seizure; JME, Juvenile Myoclonic Epilepsy; SD, standard deviation; y, years.

Community control participants with no history of neurological or psychiatric diagnoses from London, UK were also recruited during the same period and completed the BIS‐brief. Controls were group‐matched to the JME cohort by age and gender.

### Clinical data assurance

We uploaded JME clinical data onto a secure central database where a phenotyping panel, comprising seven epilepsy experts (CPB, KH, DKP, MPR, GR, MS, RT) confirmed the diagnosis of JME according to inclusion criteria. Disagreements were resolved through consensus.

### Seizure types and frequency

JME participants reported whether they had experienced absence or generalized tonic‐clonic seizures (GTCS), as well as their pre‐diagnostic and current frequency of myoclonic and absence seizures, categorized as at least daily, at least weekly, less than weekly or none. Participants also reported the number of GTCS experienced pre‐diagnosis, whether their myoclonic seizures had a morning predominance and approximate ages of seizure onset and JME diagnosis.

### Antiepileptic drug therapy

Participants reported their lifetime use of four common AEDs used to treat JME (valproate, lamotrigine, levetiracetam, and topiramate) and their experience of six adverse reactions associated with the same AEDs; (a) weight gain on valproate; (b) a skin rash requiring drug withdrawal on lamotrigine; (c) any psychiatric adverse event to levetiracetam; (d) weight loss on topiramate; (e) any psychiatric adverse event to topiramate; and (f) any cognitive adverse event to topiramate.

### Barratt Impulsiveness Scale (BIS)

**Figure 1:**
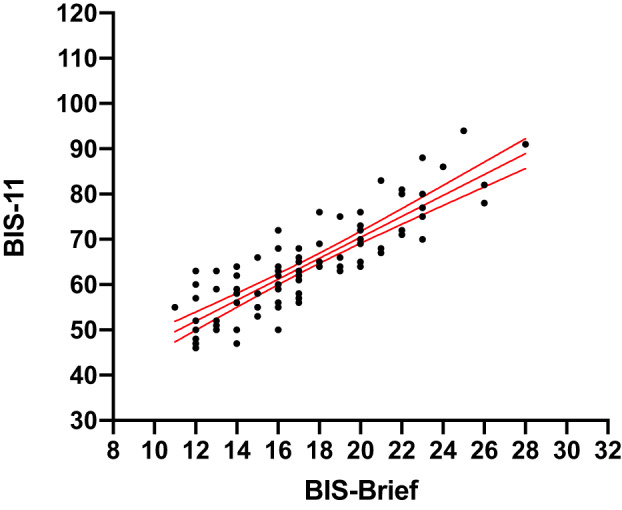
The correlation between BIS‐brief and BIS‐11 scores in 92 JME participants. The solid line is the regression line (y = 24.2 + 2.3*X). Blue dashed lines show 95% confidence intervals of regression line. R_S_ = 0.85.

We used the BIS‐brief to measure trait impulsivity in JME and control cohorts. The BIS‐brief is a shortened eight‐item version of the 30‐question BIS‐11^29^ developed by Steinberg et al.^30^ The original BIS‐11 is well‐established and cross‐validated by clinical and experimental observations.^29^, ^31^ We chose the BIS‐brief due to data available from normative and neuropsychiatric groups for comparison (see Table 2), in addition to ease of administration in a large‐scale international study. Also, BIS‐brief shows high test–retest reliability across both short and long timescales and concurrent validity with other impulsivity measures.^32^ The maximum BIS‐brief score (32) indicates high impulsivity and the minimum score (8) indicates low impulsivity. Of the 322 JME individuals who completed the BIS‐brief, 92 Norwegian participants also answered the full BIS‐11. The validity of the BIS‐brief in JME was compared to the BIS‐11, using a Spearman’s correlation. There was a strong correlation between the two scores (Fig. 1, r_s_ = 0.85, *P* < 0.001).

**Table 2:** Demographic information and mean BIS‐brief and BIS‐11 scores of JME, control and clinical comparison samples.

BIS‐brief	Study	N	Population	Female (%)	Age range (y)	Mean BIS‐brief score	SD
	Steinberg et al. (2013)^30^	128	Female controls	100%	18‐63	13.49	3.09
	Mathias et al. (2018)^32^	356	Youth controls	57%	10‐17	14.87	3.55
	**Current study**	**126**	**Control**	**58%**	**14‐66**	**16.19**	**4.06**
	Mathias et al. (2018)^32^	302	Community youth with a family history of substance use disorder, but who had not initiated regular substance use themselves.	50%	10‐12	16.35	4.27
	Steinberg et al. (2013)^30^	111	Men recently convicted of domestic violence attending an intervention program.	0%	18‐71	16.4	4.6
	**Current study**	**322**	**JME**	**67%**	**11‐53**	**18.07**	**4.44**
	Mathias et al. (2018)^32^	322	Adolescents who had recently received inpatient psychiatric care.	51%	12‐17	21.29	4.42

BIS‐11	Study	N	Population	Female (%)	Age (y ± SD)	Mean BIS‐11 score	SD
	Jepsen et al. (2018)^35^	45	Control	60%	15.5 ± 1.4	56.3	9.2
	Del Carlo et al. (2011)^34^	45	Control	62%	34.8 ± 10.2	57.4	7.6
	Malloy‐Diniz et al. (2007)^36^	51	Control	39%	32.2 ± 12.9	59.4	13.3
	Jeyadevan et al. (2019)^37^	302	Parkinson’s Disease	38%	64.4 ± 9.2	59.7	9.33
	Nandagopal et al. (2011)^41^	25	Control	44%	15.3 ± 1.7	62.1	10.7
	Tan et al. (2015)^39^	83	OCD	52%	30.1 ± 9.87	62.2	9.19
	Stanford et al. (2009)^31^	1577	Control	75%	17‐45^a^	62.3	10.3
	Riley et al. (2018)^38^	87	Parkinson’s Disease	38%	62.8 ± 9.2	62.5	8.9
	Del Carlo et al. (2011)^34^	47	Anxiety disorder	64%	34.5 ± 10.3	64.4	8.7
	**Current study**	**92**	**JME**	**61%**	**25.7 ± 6.9**	**64.9**	**10.2**
	Fossati et al. (2015)^40^	217	Personality disorder	55%	39 ±10.7	66.7	10.48
	Nandagopal et al. (2011)^41^	30	ADHD	33%	14.9 ± 1.9	71.5	12.1
	Nandagopal et al. (2011)^41^	31	Bipolar Disorder	45%	15.3 ± 1.5	75.1	8.1
	Jepsen et al. (2018)^35^	29	First‐episode schizophrenia spectrum disorders	72%	16 ± 1.2	75.4	11.8
	Malloy‐Diniz et al. (2007)^36^	50	ADHD	44%	33.7 ± 11.7	77.3	10.8
	Jepsen et al. (2018)^35^	29	ADHD	48%	15.4 ± 1.4	81.7	11.3

Note:

Rows in bold are cohorts collected as part of the current study.

Abbreviations: ADHD, attention‐deficit/hyperactive disorder; JME, Juvenile Myoclonic Epilepsy; OCD, Obsessive Compulsive Disorder; SD, standard deviation; y, years.

^a^ No mean age provided so range given.

### Analysis procedure and statistical methods

We carried out statistical analysis on SPSS statistics (version 25) and produced graphics using GraphPad Prism (version 8.4.2). We first tested all statistical assumptions, and if necessary, transformed data as appropriate or used equivalent non‐parametric tests. Individuals with missing/unknown data for specific variables were excluded from each applicable analysis. We used the following tests: Chi‐squared test for categorical variables; Spearman’s correlation to test associations between two ordinal variables; and Student’s t‐test or Mann–Whitney test for comparison of continuous variables between two groups.

We calculated a mean BIS‐brief score (± standard deviation) for the JME cohort and compared with control and neuropsychiatric samples (described in Table 2) using a pairwise one‐way Welch’s analysis of variance (ANOVA) with Games‐Howell correction (using GraphPad Prism). To compare with a broader range of clinical cohorts from the literature, we also calculated a mean BIS‐11 score from the subset of 92 individuals who completed the full BIS‐11 and observed its rank in context with other cohorts outlined in Table 2.

To test associations of clinical variables with BIS‐brief score in JME, we first performed univariate analysis (correlations for continuous data and t‐tests or ANOVAs, or non‐parametric equivalents, for categorical variables) and calculated effect sizes. Hedge’s g was used as sample sizes were unequal for all comparisons. We investigated confounding by gender in each univariate analysis by observing the change in regression coefficient when gender was added to a linear regression of BIS‐brief score with each variable. The following variables were used in a multiple linear regression model of BIS‐brief score: current frequency of absence seizures (*daily, weekly, less than weekly, none*); current frequency of myoclonic seizures (*daily, weekly, less than weekly, none*); gender *(male, female);* body mass index (BMI); morning predominance of myoclonus (*yes, no*); topiramate use (*yes, no*); psychiatric adverse reaction to levetiracetam (*yes, no*).

To investigate whether highly impulsive participants are at increased risk of a psychiatric adverse event to levetiracetam, we performed a logistic regression of current frequency of absence and myoclonic seizures, gender, BMI, morning predominance of myoclonus, and high impulsivity, against experiencing any psychiatric adverse event to levetiracetam. High impulsivity was defined as BIS‐brief ≥ 21, interpreted from the classification of high impulsivity from Stanford et al.^31^ where BIS‐11 scores ≥ 72 are considered highly impulsive. Using results from a linear regression of BIS‐11 and BIS‐brief scores (regression line: BIS‐11 = 24.2 + 2.3*BIS‐brief, Fig. 1), a BIS‐brief score of 21 is equivalent to a BIS‐11 score of 72.

### Ethics and funding

BIOJUME is funded by the Canadian Institutes for Health Research (MOP‐142405) and received ethical approval from the National Health Service (NHS) Health Research Authority (South Central ‐ Oxford C Research Ethics Committee, reference 16/SC/0266) and the Research Ethics Board of the Hospital for Sick Children, Toronto (REB# 1000033784). Local ethical approvals were also held for all international sites. All procedures complied with appropriate regulatory requirements and ethical principles in line with the Declaration of Helsinki. Informed consent was obtained and documented for all participants. Assent was obtained from minors (under 16), and informed consent was obtained on their behalf by a parent or legally appropriate guardian. All clinical data from participants were de‐identified before entry onto the central database.

## Results

### Demographics and clinical characteristics of JME cohort

#### Eligibility and general demographics

322 JME participants completed the BIS‐brief. General demographics and clinical information are presented in Table 1. The female bias, well‐known in JME, was observed (2F:1M). The median BMI was 23.7 kg/m^2^ (range 15‐52); with 43.2% of cases classified as overweight or obese. 93% of participants were of self‐reported European ethnicity.

#### Seizure characteristics

The median age of myoclonic seizure onset was 14.4 years (range 6–25), and the median age of JME diagnosis occurred around one year later at 16.1 years (range 6.9–40.5). Among those who reported other seizure types, the median age of GTCS onset was 15.4 years (range 5.7–37.8) and the median age of absence seizure onset was 6.1 years (range 3.0–11.3) in a subset of individuals who had Childhood Absence Epilepsy (CAE) evolving into JME (n = 22), and 14.2 years (range 6.9–37.1) in those who did not have CAE before a JME diagnosis (n = 131). Frequencies of seizure characteristics are presented in Table 1. We see a significant association between having daily myoclonic seizures and daily absence seizures (*P* = 5x10^‐8^).

#### Antiepileptic drug therapy

We found significant gender differences in AED therapy, with lower use of valproate and higher use of lamotrigine and levetiracetam in females (Table 1). This disparity between AED use in males and females is likely due to the revision of prescribing guidelines for valproate in females of childbearing age.^33^ 42% of individuals experienced at least one severe adverse drug event, with 37% experiencing a psychiatric event to either levetiracetam or topiramate, compared to 32.3% of individuals experiencing a somatic adverse event on valproate, lamotrigine, or topiramate. Experiencing a psychiatric adverse event to levetiracetam (37%) was most common, followed by weight loss on topiramate (36%) and weight gain on valproate (33%). More females reported weight gain on valproate than males (40% female vs. 24% male, *P* = 0.014).

### Impulsivity

**Figure 2:**
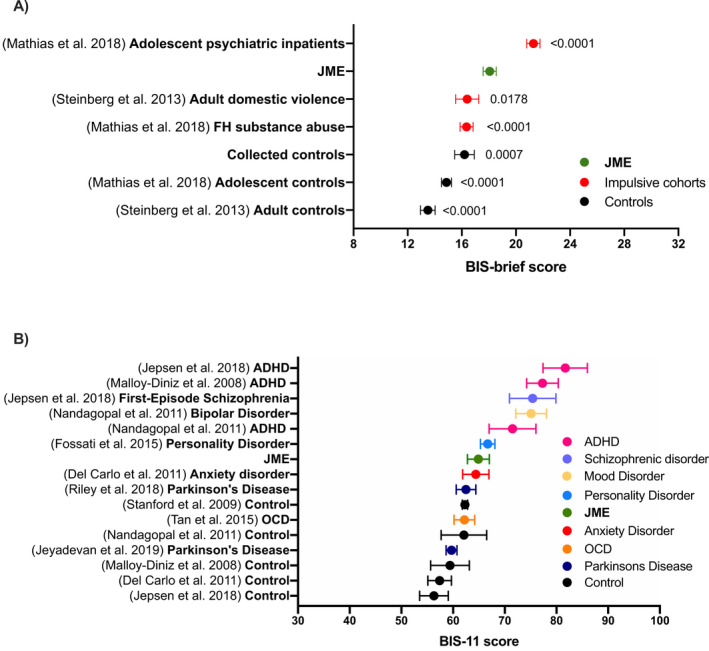
A, The mean BIS‐brief scores in our JME and control cohorts compared to other neuropsychiatric and control populations from the literature. Adjusted p‐values comparing the mean score from each group to the JME cohort are presented. Adolescent controls n = 356; adult controls n = 128; adult domestic abusers n = 111; Family history (FH) of substance abuse n = 302; adolescent psychiatric inpatients n = 322; JME n = 322; collected controls n = 126. B, Mean BIS‐11 scores of a range of control and clinical cohorts from previous literature categorized by disease type. Error bars show 95% confidence intervals. (ADHD, Attention‐deficit/hyperactive disorder; JME, Juvenile Myoclonic Epilepsy; OCD, Obsessive Compulsive Disorder).

The mean JME BIS‐brief score (18.1 ± 4.4) was significantly higher than in matched controls (16.2 ± 4.1) (*P* = 0.0007, Hedges’ g = 0.43). Cohorts did not differ significantly in age or gender (JME: median age 24, 67% female; control: median age 21, 58% female). BIS‐brief scores from all other control and outpatient cohorts (all except adolescent psychiatric inpatients), were significantly lower than the mean JME BIS‐brief score (Fig. 2A, Table 2).

The mean BIS‐11 score in a subset of the JME cohort (n = 92) was compared to other clinical and control cohorts (Table 2, Fig. 2B). There was no difference between BIS‐brief score (p = 0.39), age (p = 0.56) or gender (p = 0.27) in this subset of individuals compared to the full JME cohort. The mean JME BIS‐11 score was 64.9 ± 10.2, and therefore higher than control,^34^, ^35^, ^36^ Parkinson’s disease (PD)^37^, ^38^ and obsessive‐compulsive disorder (OCD),^39^ coinciding with personality^40^ and anxiety disorder,^34^ and lower than ADHD,^35^, ^36^, ^41^ schizophrenia^35^ and mood disorder^41^ cohorts (Fig. 2B).

To test clinical associations of trait impulsivity, we performed exploratory univariate analysis of demographic and clinical variables with BIS‐brief scores. Effect sizes and p‐values are shown in Table 3, with the largest effect size existing between individuals who are currently not having absence seizures (lower BIS‐brief) compared to those having daily absence seizures (higher BIS‐brief) (Hedges’ g = 1, 95% CI: 0.56‐1.44). There was no significant effect of age, gender, duration of JME, GTCS, AED use, adverse reactions to topiramate, or any adverse somatic event (Table 3).

**Table 3:** P‐values and effect sizes of univariate tests of BIS‐brief scores with clinical variables. For direction of changes for significant variables, see Fig. 3.

Variable	Test	*P* value	Effect size (Hedges’ g)	Effect size (g) 95% CI
Current freq. of absence seizures (none vs. daily)	Mann‐Whitney	**0.00006**	1.00	0.56–1.44
Current freq. of myoclonic seizures (none vs. daily)	T‐test	**0.001**	0.72	0.29–1.15
Topiramate use (yes vs. no)	Mann‐Whitney	**0.01**	0.43	0.12–0.74
BMI (log transformed) (cont.)	Pearson correlation	**0.02**	N/A	N/A
Psychiatric adverse reaction to levetiracetam (yes vs. no)	Mann‐Whitney	**0.04**	0.30	−0.01–0.60
Sex (male vs. female)	T‐test	**0.04**	0.24	0.007–0.47
Weight loss on topiramate (yes vs. no)	Mann‐Whitney	0.06	0.67	0.05–1.30
GTCS (yes vs. no)	Mann‐Whitney	0.07	0.28	−0.06–0.63
Morning predominance of myoclonus (yes vs. no)	Mann‐Whitney	0.19	0.20	−0.06–0.47
Levetiracetam use (yes vs. no)				
Female	Mann‐Whitney	0.35	0.13	−0.16–0.42
Male	Mann‐Whitney	0.07	0.36	−0.04–0.76
Age (log transformed) (cont.)	Pearson correlation	0.35	N/A	N/A
Psychiatric adverse reaction on topiramate (yes vs. no)				
Female	Mann‐Whitney	0.35	0.81	−0.76–3.26
Male	Mann‐Whitney	0.91	0.13	−1.40–1.66
Lamotrigine use (yes vs. no)				
Female	Mann‐Whitney	0.48	0.09	−0.20–0.39
Male	T‐test	0.55	0.12	−0.27–0.50
Skin rash on lamotrigine (yes vs. no)				
Female	Mann‐Whitney	0.50	0.28	−0.36–0.92
Male	Mann‐Whitney		0.12	−0.81–1.05
Valproate use (yes vs. no)				
Female	Mann‐Whitney	0.68	0.04	−0.22–0.31
Male	Mann‐Whitney	0.65	0.15	−0.32–0.62
Cognitive adverse reaction on topiramate (yes vs. no)	Mann‐Whitney	0.78	0.19	−0.67–1.05
JME duration (cont.)	Spearman’s rank	0.81	N/A	N/A
Weight gain on valproate (yes vs. no)				
Female	Mann‐Whitney	0.83	0.08	−0.29–0.45
Male	T‐test	0.96	0.02	−0.50–0.53

Note

Bold p‐values are significant at p < 0.05.

Abbreviations: BMI, Body Mass Index; CI, confidence interval; cont., continuous; GTCS, generalised tonic‐clonic seizures; JME, Juvenile Myoclonic Epilepsy.

**Figure 3:**
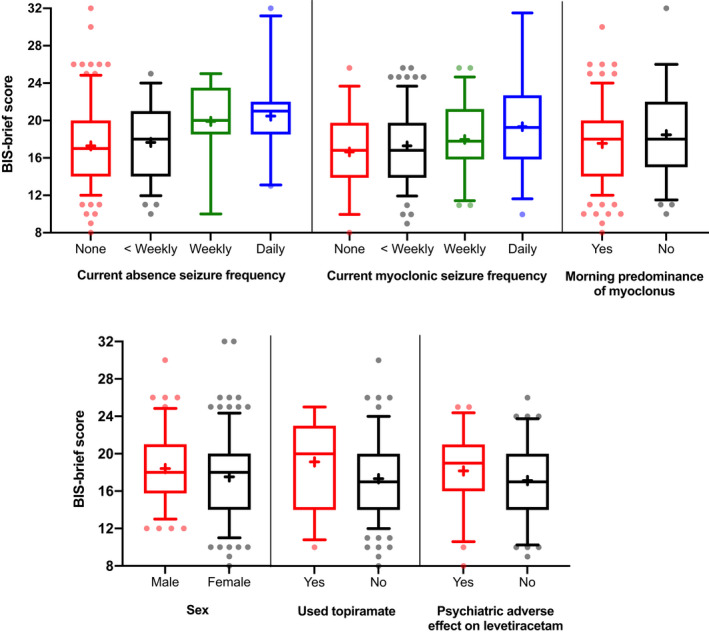
BIS‐brief score comparison between clinical variables included in the multiple linear regression model. ‘+’ shows mean per group, boxes show upper and lower interquartile range with median marked by the central line. Error bars show 95% confidence intervals with outliers presented as individual dots.

Based on these results, univariately significant variables were entered into a multiple linear regression model. Morning predominance of myoclonus was also included in the model due to an increase in adjusted r^2^ when entered (r^2^ = 0.17 vs. 0.20, p = 0.008). Therefore, the final model included: gender, current frequency of myoclonic and absence seizures, BMI, topiramate use, morning predominance of myoclonus, and having a psychiatric adverse event to levetiracetam (results presented in Table 4). The model shows an increased frequency of absence seizures is associated with increased BIS‐brief score (p = 0.0004), as is male gender (p = 0.027) and a lack of morning predominance of myoclonus (p = 0.008). Having daily absence seizures increases BIS‐brief score by a mean of 4.1 points ± 1.1 (standard error). Despite the current frequency of myoclonic seizures being significantly associated with BIS‐brief in univariate testing (Hedges’ g = 0.72) and showing a clear relationship in Figure 3, it is not a significant predictor in the regression model, likely owing to the high correlation between frequency of myoclonic and absence seizures (*P* = 1x10^‐8^). However, these variables have low collinearity in the regression model (frequency of absence seizures, tolerance = 0.8, variance inflation factor (VIF) = 1.3; frequency of myoclonic seizures, tolerance = 0.8, VIF = 1.3).

**Table 4:** Multiple linear regression of BIS‐brief score against clinical variables.

Variable	Unstandardized Coefficient		Standardized Coefficient	
	Beta	Std. Error	T statistic	*P* value
(Intercept)	13.18	4.73	2.79	**0.006**
Current frequency of absence seizures (daily, weekly, less than weekly, none)	1.36	0.37	3.65	**0.0004**
Morning predominance of myoclonus (yes, no)	−2.16	0.80	−2.70	**0.008**
Gender (male, female)	−1.95	0.87	−2.23	**0.027**
Topiramate use (yes, no)	1.72	0.92	1.87	0.063
Psychiatric adverse reaction on levetiracetam (yes, no)	1.26	0.70	1.81	0.073
BMI (log transformed) (cont.)	4.89	3.32	1.47	0.143
Current frequency of myoclonic seizures (daily, weekly, less than weekly, none)	−0.36	0.43	−0.84	0.405

*Note*

Adjusted R^2^ = 0.20, n = 148. Bold *P*‐values are significant at *P* < 0.05.

Abbreviations: BMI, Body Mass Index; cont., continuous.

Those who used topiramate showed some evidence of increased impulsivity in the linear regression model, and a small to medium effect size in univariate testing, as do those who experience a psychiatric adverse event to levetiracetam (Table 3 and 5). Figure 3 shows the distribution of BIS‐brief scores across variables included in the regression model. Logistic regression (Table 5) showed that having a BIS‐brief ≥ 21, i.e. highly impulsive, significantly increases the odds of having an adverse psychiatric event to levetiracetam by 2.3 (95% CI: 1.05‐5.00, *P* = 0.037).

**Table 5:** Logistic regression of adverse psychiatric event to levetiracetam.

Variable	Odds ratio	95% CI		Beta	Standard Error	*P* value
High impulsivity (yes, no)	2.3	1.0	5.1	0.76	0.41	**0.039**
BMI (log transformed) (cont.)	0.1	0.01	4.3	−1.94	1.74	0.259
Gender (male, female)	0.6	0.3	1.5	−0.48	0.45	0.279
Current frequency of myoclonic seizures (daily, weekly, less than weekly, none)	1.2	0.8	1.9	0.21	0.23	0.349
Morning predominance of myoclonus (yes, no)	1.4	0.6	3.2	0.31	0.43	0.469
Current frequency of absence seizures (daily, weekly, less than weekly, none)	1.0	0.7	1.5	0.003	0.20	0.869
(Constant)	6.5	0.05	814.4	1.81	2.46	0.446

Note

Nagelkerke R^2^ = 0.08, n = 148. Bold *P*‐values are significant at *P* < 0.05.

Abbreviations: BMI, Body Mass Index; CI, confidence interval; cont., continuous.

## Discussion

Trait impulsivity is significantly elevated in JME over matched controls by two points on the BIS‐brief and to a score comparable to personality and neurotic disorders in the literature using BIS‐11.^34^, ^39^, ^40^ The extent of this psychological comorbidity in epilepsy has previously not been well‐defined, and adds to known associations of impulsivity with personality disorders and externalizing psychiatric disorders.^9^, ^22^, ^23^, ^30^, ^32^, ^42^ Furthermore, our results substantiate evidence of elevated response and choice impulsivity in JME through experimental tasks,^5^, ^10^, ^11^, ^12^, ^13^, ^14^ suggesting a robust elevation in broad impulsivity. The association with seizure frequency and absent circadian pattern of seizures, points to possible mechanisms involving frontostriatal and thalamocortical networks, suggesting hypotheses for intervention. The increased odds of a psychiatric adverse event to levetiracetam in those with elevated impulsivity supports previous findings^19^ and possibly reflects a genetic predisposition.

### Clinically significant elevated impulsivity

Trait impulsivity is significantly higher in individuals with JME compared to matched controls, with a mean 2‐point score difference and medium effect size. A BIS‐11 score of 72 is a suggested cut‐off for severity,^31^ therefore, stratifying 24% of the current sample (27% if the BIS‐brief equivalent of 21 is used). The clinical severity of this trait is also contextualized by comparison with other samples. BIS‐brief scores in JME were higher than in cohorts at risk of psychopathology,^30^, ^32^ and BIS‐11 scores were higher than PD^37^, ^38^ and OCD samples,^39^ overlapped with anxiety and personality disorder samples^34^, ^40^ and lower than mood and schizophrenia spectrum disorder^35^, ^40^, ^41^ and ADHD samples.^35^, ^36^, ^41^ This extends findings from smaller, uncontrolled studies of trait impulsivity in JME using the BIS‐11^12^ and the Temperament and Character Inventory,^10^, ^13^ and provides robust evidence for associations across multiple dimensions of impulsivity, including response and choice impulsivity.^5^, ^9^, ^14^ We recently reported a moderate and homogeneous effect size (d = 0.50, 95% CI 0.37‐0.63) for response inhibition in a meta‐analysis of 1047 JME patients.^9^ Similarly, with choice impulsivity, JME patients, especially those with persistent seizures, have difficulty learning advantageous decisions in the Iowa Gambling Task (IGT), while seizure‐free patients performed equal to controls.^5^, ^14^ Although our cross‐sectional design does not allow inference about directionality, functional imaging studies support the interpretation that either seizures result in increased trait impulsivity or loss of striato‐cortical inhibition influences both seizure susceptibility and impulse control.^3^, ^4^, ^14^ A further limitation of the study design is the potential of ascertainment bias in both JME and control cohorts. The influence of an individual’s impulsiveness on participating in a study such as this is unknown, however, this would likely influence both JME and control cohorts similarly. Conversely, potential ascertainment biases differing between JME and control cohorts may include educational level, socio‐economic background, and ethnicity which were not controlled for. However, we aimed to address this limitation by using a range of control cohorts from previous literature alongside our own age and gender‐matched controls, all of which have lower impulsivity than the JME cohort.

Impulsivity influences treatment outcome through reduced treatment adherence in substance abuse^22^ and gambling disorders.^23^ Although we were unable to test the association here, adherence to lifestyle modifications, including limiting alcohol and maintaining a stable sleep routine, in addition to regular AED use, is essential to JME seizure management.^28^ The link to social prognosis requires further investigation, but problem behaviors associated with impulsivity in the population, including substance abuse, violence and illegal activities such as shoplifting or reckless driving,^43^ closely resembles findings of two population‐based interview studies of psychosocial outcome in JME patients.^24^, ^25^ In both studies, there was no relationship between seizure and social outcome, which underlines the value of longitudinal investigations of impulsivity and associated psychiatric features as long‐term risk factors.

### Moderators of impulsivity

Seizure frequency is the strongest predictor of trait impulsivity, with effect sizes ranging from 0.72 to 1.00 for daily versus no current myoclonic or absence seizures, and a clear dose‐response relationship. By analogy, the four‐point rise in BIS‐brief with daily absence seizures is equivalent to the difference between asymptomatic individuals’ and psychiatric inpatients’ mean scores.^32^ However, seizure‐free patients also had an elevated mean BIS‐brief score compared to controls. Absence seizures demonstrate the strongest effect on impulsivity compared to myoclonic or GTC seizures, possibly reflecting additional disruption to attention and impulse control networks.^44^ The effect size, though large, is possibly an underestimate due to notorious under‐reporting of absence seizures. A previous study showed that active myoclonic seizures rather than active absence seizures were associated with an increased BIS‐11 score in a genetic generalized epilepsy (GGE) cohort,^15^ however, no interaction analysis between GGE subtype and the effect of active absence seizures on BIS‐11 was performed therefore preventing a direct comparison to results from this study of JME. Other studies demonstrate that seizure frequency also moderates choice impulsivity,^5^, ^14^ but there were insufficient data to explore this in response impulsivity.^9^

A circadian pattern or morning predominance of seizures is a touchstone of JME diagnosis and enshrined in consensus criteria.^28^ Loss of this circadian pattern was associated with a small but significant effect size in trait impulsivity. Loss of morning predominance may be associated with unfavorable prognosis as it is more often found in JME patients with absence seizures or with worse overall seizure control, putatively because of disruption of circadian thalamocortical oscillators.^45^ However, the association with trait impulsivity remains even after controlling for absences, suggesting residual confounding (unreported absences), heterogeneity or confounding by sleep. A poor sleep routine affects attention, executive function, and memory in healthy individuals^46^ and those with high BIS‐11 scores have phase‐delayed sleep patterns, decreased total sleep time and efficiency and disrupted circadian function.^47^ Since sleep fragmentation triggers seizures in JME, a poor sleep routine may confound this association, causing both increased seizure frequency and increased impulsivity.

The extent to which impulsivity could be reduced by improved seizure control remains an open question. JME is usually managed by AEDs with broad efficacy for generalized seizure types,^21^ but it is possible that complementing these with an absence seizure‐specific AED (e.g. ethosuximide) may reduce impulsivity. However, complete seizure control is not a feasible goal for 20% of patients,^24^ therefore warranting consideration of alternative approaches to reducing impulsivity, such as cognitive‐behavioral therapy.^48^ The complicated relationship between sleep and impulsivity in epilepsy may also yield testable hypotheses for intervention.

Elevated broad impulsivity in JME may partly be attributable to its adolescent‐onset and partly to the pathophysiology of JME, which involves disruption to normal development and function of both frontostriatal and thalamocortical structures and respective connections.^2^, ^3^, ^4^, ^5^, ^6^ There is convergent evidence for abnormalities in frontostriatal and thalamocortical networks in JME, drawn from neuroimaging and neuropsychological studies.^2^, ^3^, ^6^, ^49^ Taken together, there is considerable overlap in prefrontal, striatal, and limbic networks involved in both JME and impulse control. Other epilepsies may also be associated with raised impulsivity but are less studied.^8^, ^44^

### Treatment outcome

Neuropsychiatric adverse events were reported in over one‐third of participants and were more common than somatic adverse events. We observed a two‐fold increase in the odds of a psychiatric adverse reaction to levetiracetam in highly impulsive individuals compared to less impulsive individuals. There was no association of impulsivity with any adverse event to topiramate, nor valproate or lamotrigine use, nor any somatic adverse effects to these AEDs. These data alone are not conclusive, partly due to absent baseline impulsivity data before levetiracetam exposure and the inability to exclude potential effects of current AED treatment on BIS scores, since information on lifetime therapy was provided rather than current therapy. However, other studies show similar findings. Helmstaedter et al.^19^ showed that individuals with epilepsy and high BIS‐11 scores were more likely to suffer from adverse psychiatric effects to levetiracetam. A model used to predict this adverse effect showed that individuals with a history of neuropsychiatric disorders, including depression, anxiety, personality disorder, or recreational drug use (all outcomes associated with impulsivity), were at increased risk.^20^ Other studies show that individuals with epilepsy and a history of behavioral or psychiatric conditions were more likely to experience a psychiatric side effect across many AEDs, not only levetiracetam.^18^, ^50^ Additionally, we must consider a potential genetic predisposition to psychiatric adverse events to levetiracetam in those individuals who are more impulsive.^26^, ^27^ Dopamine receptor alleles are suggested in a candidate gene study,^26^ but replication is necessary.

Finally, there is suggestion of increased impulsivity with topiramate use. However, exposure to topiramate, which is a second or third‐line AED for JME,^21^ may be a proxy for poor seizure control or drug‐resistance, rather than representing a sign of chronic topiramate neurotoxicity.

## Summary and conclusions

Multiple dimensions of raised impulsivity are associated with JME. The robust association of trait impulsivity with seizure frequency and loss of circadian pattern implies dysfunction in prefrontal, striatal, and limbic brain networks. Improved seizure control, perhaps complemented by psychotropic or cognitive‐behavioral interventions, may be considered to mitigate this psychological comorbidity. Whether these findings also apply to other epilepsy syndromes,^8^ as well as the implications of impulsivity for AED adherence and psychosocial prognosis in JME remain unexplored. We demonstrate that the BIS‐brief, a simple measure of trait impulsivity, may have utility in predicting adverse psychiatric effects to levetiracetam. Genetic mediators of this risk would offer an advance in precision medicine. Overall, these findings demonstrate the importance of looking beyond diagnostic categories toward endophenotypes that better capture the components influencing patient‐relevant outcomes and allow the development of better‐targeted treatments.

## Author Contributions

AS, NP, CPB, KH, JK, MR, GR, KKS, ABS, PS, MS, RT, MPR, LJS, and DKP contributed to conception and study design. AS, NP, RM, HC, and DKP contributed to data management and project administration. DA, JPC, CPB, CYF, JG, DAG, KH, KSL, JK, AO, MR, KKS, MS, IT, RT, JZ, MPR, and DKP contributed to acquisition of study data. AS, NP, LJS, and DKP contributed to analysis of data. AS, NP, LJS, and DKP contributed to drafting the manuscript. Members of the BIOJUME consortium are listed in the appendix.

## Conflicts of Interests

DA, KKS, RT, and JZ report honoraria from UCB Pharma (manufacturer of levetiracetam) and RT reports honoraria from Sanofi (manufacturer of sodium valproate). All other authors report no conflicts of interest.

## BIOJUME consortium

**Canada:** Lisa Strug, Hospital for Sick Kids; Danielle Andrade, Toronto Western Hospital; **Czech Republic**: Jana Zarbubova, Charles University; **Denmark:** Guido Rubboli, Rikke Moller & Elena Gardella, Danish National Epilepsy Centre; Christoph P. Beier & Joanna Gesche, Odense University Hospital; **Estonia:** Inga Talvik, Tallinn Children’s Hospital; **France:** Stéphane Auvin, University Robert Debré; **Italy**: Alessandro Orsini & Pasquale Striano, Commissione Genetica Lega Italiana Contro l’Epilessia; Andrea Praticò, Università degli Studi di Catania; **Malaysia:** Choong Yi Fong, Kheng Seang Lim & Ching Ching Ng, University of Malaya; **Norway:** Jeanette Koht, Kaja K. Selmer & Marte Syvertsen, Vestre Viken Health Trust; **United Kingdom:** Pronab Bala & Amy Kitching, Airedale NHS Foundation Trust; Kate Irwin, Lorna Walding & Lynsey Adams, Ashford and St. Peter’s Hospitals NHS Foundation Trust; Dan Hindley, Joanne Henry, & Claire Abbott, Bolton NHS Foundation; Uma Jegathasan, Rachel Wane & Rachel Swingler, Bradford Teaching Hospitals NHS Foundation Trust; Julia Aram, Nikil Sudarsan, Dee Mullan, Rebecca Ramsay & Vivien Richmond, Brighton and Sussex University Hospitals NHS Trust; Matthew Taylor, Susan Kilroy, Tonicha Nortcliffe & Marie Home, Calderdale & Huddersfield Foundation Trust; Khalid Hamandi & Alison McQueen, Cardiff & Vale University Health Board; Hannah Steele & Andrew Smith, City Hospitals Sunderland NHS Foundation; Dina Jayachandran & Dawn Egginton, County Durham and Darlington NHS Foundation Trust; Bridget MacDonald, Croydon Health Services NHS Trust; David Deekollu, Alok Gaurav & Caroline Hamilton, Cwm Taf Morgannwg University Health Board; Shane Delamont, Dartford and Gravesham NHS Trust; Inyan Takon, East and North Hertfordshire NHS Trust; Nick Moran, East Kent Hospitals University NHS Foundation Trust; Rosemary Belderbos, Heather Collier & Joanne Henry, East Lancashire Hospitals NHS Trust; Michalis Koutroumanidis & Javier Peña Ceballos, Guy’s and St Thomas’ NHS Foundation Trust; Mark P. Richardson, Jennifer Quirk & Javier Peña Ceballos, King’s College Hospital NHS Foundation Trust; Dora Lozsadi, Kingston Hospital NHS Foundation Trust; Tahir Majeed, Janice Birt & Sonia Raj, Lancashire Teaching Hospitals NHS Foundation Trust; Melissa Maguire, Munni Ray, Caroline Peacey, Linetty Makawa & Asyah Chhibda, Leeds Teaching Hospitals NHS Trust; Lap Yeung, Claire Holliday & Louise Woodhead, Manchester University NHS Foundation Trust; Rhys Thomas, Shan Ellawela, Joanne Glenton & Verity Calder, Newcastle upon Tyne Hospitals NHS Foundation Trust; Karen Lanyon, Graham Mackay, Coleen Thow, Elma Stephen & Margaret Connon, NHS Grampian; Martin Kirkpatrick, Susan MacFarlane & Anne Macleod, NHS Tayside; Siva Kumar & Carolyn Campbell, North Tees and Hartlepool NHS Foundation Trust; William Whitehouse, Christina Giavasi, Boyanka Petrova & Thomas Brown, Nottingham University Hospitals NHS Trust; Seán J. Slaght, Catherine Edwards, Andrew Gribbin & Liz Nelson, Portsmouth Hospitals NHS Trust; Heather Angus‐Leppan, Loveth Ehiorobo & Bintou Camara, Royal Free London NHS Foundation Trust; Rajiv Mohanraj & Vicky Parker, Salford Royal NHS Foundation Trust; Rajesh Pandey, Lisa Charles & Catherine Cotter, Sandwell & West Birmingham Hospitals NHS Trust; Archana Desurkar & Alison Hyde, Sheffield Children’s NHS Foundation Trust; Markus Reuber, Rosie Clegg, Jo Sidebottom & Mayeth Recto, Sheffield Teaching Hospitals NHS Foundation Trust; Rameshb Kumar, Nikolas Hitiris & Sonia Armstrong, South Tees Hospitals NHS Foundation Trust; Shyam Mariguddi & Zena Haslam, Southport and Ormskirk Hospital NHS Trust; Hannah Cock, Mark Mencias, Samantha Truscott & Deirdre Daly, St George’s University Hospitals NHS Foundation Trust; Mark Rees, Seo‐Kyung Chung & Owen Pickrell, Swansea University Medical School and Swansea Bay University Healthboard; Amy Whiting & Kirsty O’Brien, Taunton & Somerset NHS Foundation Trust; Fraser Scott, Naveed Ghaus, Gail Castle & Jacqui Bartholomew, The Mid Yorkshire Hospitals NHS Trust; Shashikiran Sastry & Charlotte Busby, The Royal Wolverhampton NHS Trust; Amit Agrawal, Debbie Dickerson & Almu Duran, The Walton Centre NHS Foundation Trust; Muhammad Khan, Laura Thrasyvoulou, Eve Irvine, Sarah Tittensor & Jacqueline Daglish, University Hospitals Birmingham NHS Foundation Trust; Sumant Kumar & Claire Backhouse, University Hospitals of Derby and Burton NHS Foundation Trust; Anas Olabi & Kathryn Allison, University Hospitals of Morecambe Bay NHS Foundation Trust; Rahul Bharat, Sarah‐Jane Sharman & Debbie Coker, University Hospitals Plymouth NHS Trust; Darwin Pauldhas, Sharon Kempson & Lisa Richardson, Walsall Healthcare NHS Trust; Arun Saraswatula & Helen Cockerill, West Suffolk NHS Foundation Trust; **USA:** Rachit Patel, Hasbro Children’s Hospital; David A. Greenberg, Nationwide Children’s Hospital.
